# A 3D Faraday Shield for Interdigitated Dielectrometry Sensors and Its Effect on Capacitance

**DOI:** 10.3390/s17010077

**Published:** 2016-12-31

**Authors:** Alex Risos, Nicholas Long, Arvid Hunze, Gideon Gouws

**Affiliations:** 1 School of Chemical and Physical Sciences, Victoria University of Wellington, Wellington 6012, New Zealand; 2 Robinson Research Institute, Victoria University of Wellington, Lower Hutt 5010, New Zealand; nick.long@vuw.ac.nz (N.L.); arvid.hunze@vuw.ac.nz (A.H.); 3 School of Engineering and Computer Sciences, Victoria University of Wellington, Wellington 6012, New Zealand; gideon.gouws@vuw.ac.nz

**Keywords:** shielding, guard, noise reduction, electric field distribution, Green’s function, FEM, interdigitated dielectrometry sensor, co-planar electrodes, capacitance measurement

## Abstract

Interdigitated dielectrometry sensors (IDS) are capacitive sensors investigated to precisely measure the relative permittivity ($\epsilon_{r}$) of insulating liquids. Such liquids used in the power industry exhibit a change in $\epsilon_{r}$ as they degrade. The IDS ability to measure $\epsilon_{r}$ in-situ can potentially reduce maintenance, increase grid stability and improve safety. Noise from external electric field sources is a prominent issue with IDS. This paper investigates the novelty of applying a Faraday cage onto an IDS as a 3D shield to reduce this noise. This alters the spatially distributed electric field of an IDS affecting its sensing properties. Therefore, dependency of the sensor’s signal with the distance to a shield above the IDS electrodes has been investigated experimentally and theoretically via a Green’s function calculation and FEM. A criteria of the shield’s distance *s* = $s_{0}$ has been defined as the distance which gives a capacitance for the IDS equal to $1\;-\;e^{-2}=86.5\%$ of its unshielded value. Theoretical calculations using a simplified geometry gave a constant value for $s_{0}$/λ = 1.65, where λ is the IDS wavelength. In the experiment, values for $s_{0}$ were found to be lower than predicted as from theory and the ratio $s_{0}$/λ variable. This was analyzed in detail and it was found to be resulting from the specific spatial structure of the IDS. A subsequent measurement of a common insulating liquid with a nearby noise source demonstrates a considerable reduction in the standard deviation of the relative permittivity from $\sigma_{\mathrm{unshielded}}=\pm$9.5% to $\sigma_{\mathrm{shielded}}=\pm$0.6%. The presented findings enhance our understanding of IDS in respect to the influence of a Faraday shield on the capacitance, parasitic capacitances of the IDS and external noise impact on the measurement of $\epsilon_{r}$.

## 1. Introduction

**Figure 1:**
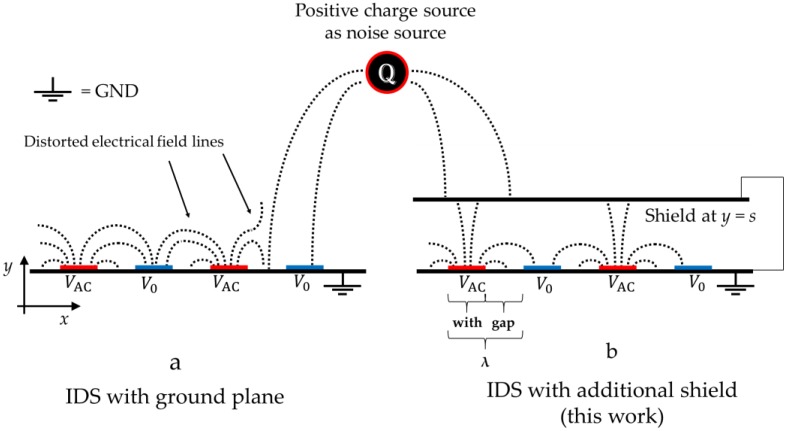
Working principle of a grounded shield. The arbitrarily positive source charge as noise source emanates an electric field which interacts with the IDS on the left hand side (**a**) whereas the shielded right hand side remains undisturbed from the external noise (**b**).

Insulating liquids used in the power industry for cooling energized conductors have been investigated regarding their properties and potential for many decades [1,2,3]. It is well known that degradation of these liquids results in a change in their static permittivity that can be detected via a capacitive measurement. Interdigitated Dielectrometry Sensors (IDS) are capacitive sensors [4,5] which can sense this permittivity in-situ via an emanated electric field [6,7]. This potentially makes IDS valuable for reducing maintenance, increasing grid stability and safety. Usually, the periodicity of the interdigitating electrodes is expressed in units of λ, where λ/2= width of electrodes + gap between the electrodes, which is proportional to the characteristic penetration depth of the emanated electric field [8,9], as shown in Figure 1.

The versatility of IDS has led to various applications such as chemically or physically active sensors. Chemically sensitive IDS, for instance, can be made by attaching a sensitive coating onto the surface within the electric field enabling a large measurable gain in the signal [7,10,11,12,13,14]. Physically sensitive IDS however, consist of uncoated electrodes to sense the permittivity of the material under test in high frequency measurements [15]. Commercial solutions such as N1501A Dielectric Probe Kit (Keysight Technologies, Santa Rosa, CA, USA) are readily available to investigate e.g., water based liquids showing a high permittivity, using electrodes in MHz to GHz domain. 

However, not much research has been done on measuring signals with uncoated IDS exhibiting low signal to noise ratio at high confidence. The measurement of the relative permittivity of insulating liquids in the presence of external electric fields is a good example of dealing with IDS measurements in a difficult environment. Such measurements can be obtained by taking the ratio of the capacitance of the insulating liquid to the air capacitance as $\epsilon_{r}=\;C_{\mathrm{ins}.\;\mathrm{liquid}}{/C}_{\mathrm{air}}$. Standard procedures [16] prescribes the use of low frequencies between 40 to 62 Hz, leading to very low currents through the IDS and thus reducing the signal. Also, insulating liquids as used in power electronics applications show a low permittivity of 2.1 to 3.5 [2,17,18] (c.f. ~80 for water) contributing to a low capacitance that further reduces the signal. This permittivity, reflecting the quality of an insulating liquid, changes by about only 10% during service time, e.g., a change in $\epsilon_{r}$ from 2.1 to 2.3 [17]. This limited change and the overall reduced signal means that noise becomes a considerable issue in precisely measuring $\epsilon_{r}$ of insulating liquids with IDS. 

IDS are susceptible to noise caused by external electric fields in the environment. These external fields interact with the electric field of the electrodes. Detailed reports about noise and uncertainty is given in previous work [9,19,20]. Reducing this noise can be achieved in various ways such as averaging over long periods or improving the measurement devices. Surrounding the sensor with an electrically grounded Faraday cage, as suggested by other researchers [20], is one method to reduce such noise, without affecting other possible measures to improve precision.

The arrangement for such a shield and its function is illustrated in Figure 1. The first step is to shield an IDS in the direction below the sensor where no sensing takes place [19,20]. This can be done using a ground plane placed as close as possible to the electrodes (but without electrical contact), as shown as GND in Figure 1a. In a similar manner, a second ground plane can be applied above the sensor as shown in Figure 1b. Since this shield will also reduce the capacitance and thus the signal, the distance *s* must be chosen carefully [20]. Thus, the capacitance *C* dependent on a grounded shield above the sensor in *y*-direction at *y = s* is studied in this work, expressed as *C*(*s*).

A mechanically rigid experimental setup was used to measure the capacitance *C* while the shield at distance *s* was varied in micrometer steps. These plots are shown and compared with a theoretical calculation via a Green’s function. Finite element method (FEM) was complementarily used to verify the Green’s function solution. A comparison of experimental data and theory shows that more accurate FEM is necessary to explain the difference between experiment and theory. A subsequent experimental test of the shield’s function shows considerable noise reduction when the IDS is exposed to a representative noise source. 

This publication improves the current state of the art [5,8,9,19,20,21] of IDS by modifying an IDS with a Faraday shield. The addition of a shield to an IDS was hypothetically suggested in [20,21]. This paper will now extend this work by modelling the impact on the capacitance by the shield with the aid of Green’s function and FEM. This novel technique develops IDS for applications outside a shielded laboratory environment, potentially making IDS useful for in-situ measurements, such as in oil filled transformers or engines, thus opening new frontiers in IDS applications. 

## 2. Materials and Methods

### 2.1. Theoretical Approach via Green’s Function

**Figure 2:**
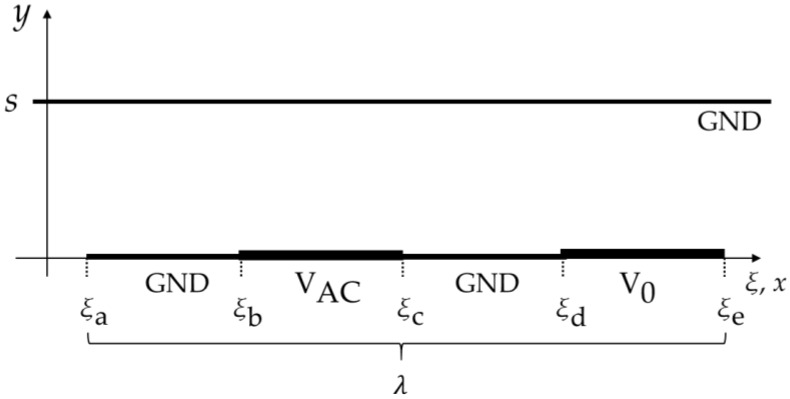
Cross section of IDS for the calculation with Green’s function. Shown is one period of *λ*.

For the calculations in the presence of the two grounded planes (GND), the IDS has been simplified to its cross sectional view as depicted in Figure 2 showing one period of λ. The shield above the electrodes is located along the *y*-axis at *y* = *s*. The space between the active and neutral electrodes, labelled $V_{\mathrm{AC}}$ and $V_{0}$, is set to V = 0 (GND), which is an approximation of having zero distance to the lower ground plane. As usually done, all geometries have no thickness and are infinite in the z-direction [22].

A Green’s function method was used to calculate the potential field and subsequently the total charge on the electrodes at $V_{0}$ to determine C(s). The problem is treated as two-dimensional by treating the *z*-direction as infinite and solving for the capacitance per unit length. This technique works by applying the boundary conditions of the problem under consideration to find the relevant Green’s function for the problem geometry. The solution to the potential is then found by an integral over the enclosing surface. General Green’s function solutions of the Laplace equation in two- dimensions are available for either infinite or finite *x* and *y*-boundaries (c.f. p. 710 in [23]). For instance, the electric field problem of an IDS with boundaries at infinity without a ground plane above and below the electrodes has been solved in a previous study [22]. The shield however introduces a new boundary problem where the *x*-direction remains infinite and *y* is limited by the shield distance. A solution was found via transforming the known series solution for finite boundaries in x and y (c.f. p. 455 in [23]):

(1) $$G\left(x,y|\xi ,s\right)=\frac{2}{a}\sum_{n=1}^{\infty }\;\frac{\sinh[(\pi n/a)(s-y)]}{\sinh\left(\pi ns/a\right)}\sin\left(\frac{\pi nx}{a}\right)\sin\left(\frac{\pi n\xi }{a}\right),$$

with ±*a* as the position of the boundary on the *x*-axis and *y* = 0, *y* = *s*, the *y*-axis boundaries, to a result valid for *a* → ±∞:

(2) $$\;G\left(x,y|\xi ,s\right)\;=\;\frac{1}{\pi }\int_{0}^{\infty }\frac{\sinh k\left(s-y\right)}{\sinh ks}\cos k(x-\xi )dk.$$

The potential Ψ(x,y|s) of the IDS with *n* periods of λ is given by the integral of the product of the Green’s function with the potential function ψ(ξ) over the ξ-space along x at y = 0:

(3) $$\Psi \left(x,y|s\right)\;=\;\underset{n}{\sum }\int_{\xi_{b}{+n\lambda }_{\;}}^{\xi_{c}+n\lambda }G\left(x,y|\xi ,s\right)\psi (\xi )d\xi .$$

The potential function ψ(ξ) is a step function with amplitude ψ(ξ)=1 V on the active electrodes and ψ(ξ)=0 V on the neutral electrodes with is equal to GND which is extended along the *x*-axis to the left and right of the calculated domain.

Employing Gauss’s law, the surface charges along x are given by the limit of the gradient of this potential function in y-direction $\sigma_{\;}(x)\;=\;\underset{y\to 0}{\lim}\frac{\partial \Psi \left(x,y|s\right)}{\partial y}\epsilon_{0}$ [24]. The capacitance C is consequently the modulus from the integral of the charges over the neutral electrode, multiplied by the actual electrode length *l* in the *z*-direction:

(4) $$C(s)\;=\;l\underset{n}{\sum }\int_{\xi_{d}+n\lambda }^{\xi_{e}+n\lambda }{|\sigma }_{x}|dx.$$

Equation (4) was solved numerically using the limits for $\underset{y\to 0}{\lim}\frac{\partial \Psi \left(x,y|s\right)}{\partial y}$ at *y* = 0.000675λ due to the mesh size in MATLAB version R2014a (this MATLAB script can be obtained directly from the corresponding author).

### 2.2. Experimental Setup and Used IDS

The employed multi-wavelength IDS board was produced via standard commercial multilayer PCB technology. This cost efficient solution was used as it allows rapid prototyping and testing of our sensor structure. The wavelengths λ, having a width/gap-ratio of 1, have been chosen as 1 mm, 2 mm and 4 mm based on previous literature [8,9,19,20,21] and considering that λ = 1 mm required a trace width of 250 µm which was a physical lower limit in standard PCB technology (Shenzhen JDB Technology Co., Ltd, Hangzhou, China). The geometry was kept as a simple square shape, the length *l* of the 20 (*n* = 10λ) electrodes were 37 mm, 18.5 mm and 9.25 mm respectively. 

**Figure 3:**
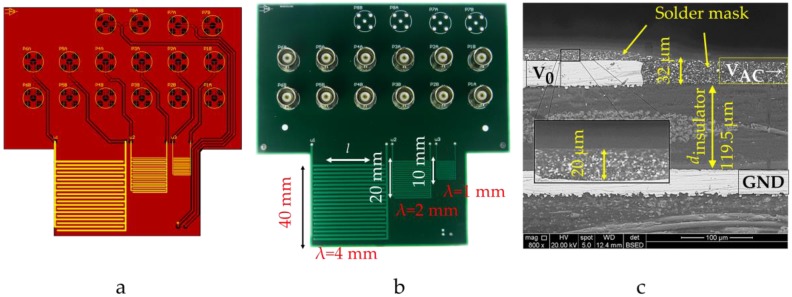
IDS used in this publication showing wavelengths and dimensions. (**a**) Multilayer PCB layout and (**b**) the manufactured version; (**c**) The SEM image shows the cross sectional structure of the manufactured IDS.

Figure 3a shows the PCB layout of the IDS depicting its multilayer structure. Here, the copper areas are color coded in red while trace separation is shown as black. The IDS electrodes are shown as yellow parallel strips. The manufactured version is shown as photograph with dimensions in Figure 3b. This multilayer board structure shields each of the four terminals with a grounded layer to the sides, below and above resulting in a 3D shielded lead design. These leads connect to the IDS electrodes through Vertical Interconnect Accesses (VIA). This approach ensures accurate and precise measurements by reducing parasitic capacitance and mutual inductance of the leads. Figure 3c depicts a cross-sectional view obtained via Scanning Electrode Microscope (SEM). Relevant dimensions are listed in Table 1.

**Figure 4:**
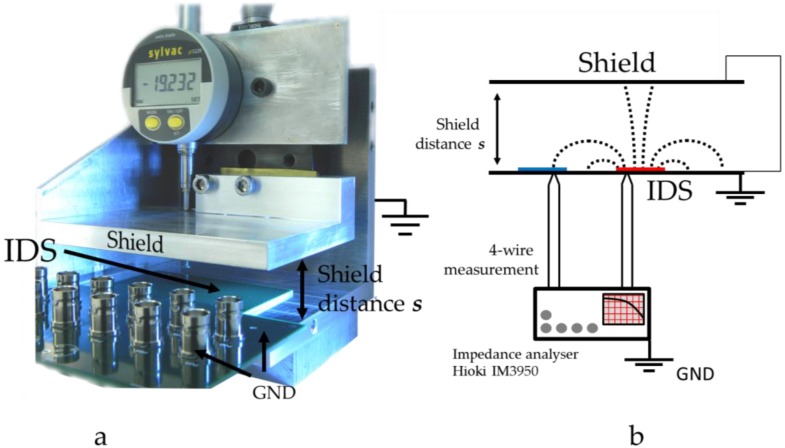
(**a**) Photo of the experimental setup. The photo was taken with an open side for visualization purposes; (**b**) Schematic of the experiment.

The experimental setup and its working principle is shown in Figure 4a,b, respectively. The machined construction covers and holds the IDS in place while allowing the shield distance *s* above to be varied. This position varied by a standard micrometer stage (PT1 from Thorlabs, Newton, NJ, USA) was read precisely via the installed digital micrometer. The rigid aluminum construction was grounded throughout the experiment to provide boundaries with ψ(ξ) = 0 V. The IDS was smaller than the grounded *xz*-plane matching the assumption made to have the ground plane extended to infinity. The measurement was carried out via a four wire capacitive measurement through our Hioki IM3950 impedance analyzer (Hioki, Nagano, Japan). This four wire setup ensured precise and accurate measurements. The grounded aluminum construction served also as a Faraday cage, so that the measurement was less affected by external noise.

## 3. Results

### 3.1. Capacitance with Shield Distance

**Figure 5:**
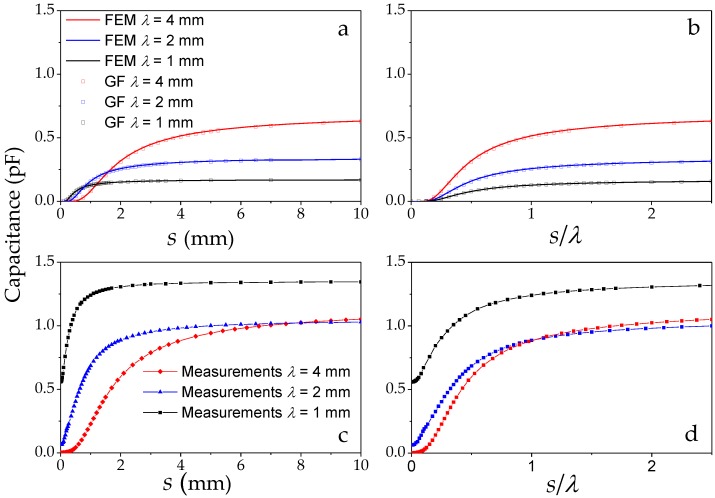
(**a**) Calculation of C(s) from (4) and agreement with FEM; (**b**) Same data plotted vs. s/*λ*; (**c**) Experimental results for three different IDS wavelengths; (**d**) Same data plotted vs. *s*/*λ*. Clearly visible is the inverted order of *s*/*λ* in the experiment due to an increased offset in the capacitance.

The *s* dependence of the capacitance calculated from Equation (4) was verified by FEM (Laplace module, Comsol Multiphysics 4.2, COMSOL, Burlington, MA, USA) applied to the same geometry as given in Figure 2. The calculations are shown in Figure 5a,b and plotted versus *s* and *s*/λ, respectively.

The capacitance of an IDS scales with the geometry parameters: length *l* in *z*-direction, width/gap-ratio, and *n*. The capacitance is independent of λ in the idealized geometry; since the width/gap ratio and the number of electrodes remained constant, only the varied length *l* changed the capacitance. Hence, the IDS, all with *n* = 10 electrode pairs, have a capacitance ratio of 1:2:4 due to the different lengths *l* in the *z*-direction. 

Figure 5b shows the capacitance versus s/λ and illustrates that, in theory, the capacitance is a function of s/λ and the magnitude is varying with *l*. For small s/λ values this figure also emphasizes a minimum s/λ distance for the shield before which the field lines cannot reach the neutral electrodes ($V_{0}$) and contribute to the capacitance. In this region, the charge on the active electrodes ($V_{\mathrm{AC}}$) is dominated by balanced charges on the upper shield.

The experimental result in Figure 5c,d shows the capacitance C(s) of each of the three sensors is much larger than the theoretical model predicts. As opposed to the prediction, the order of the capacitance magnitudes is reversed over most of the range. The measurements for wavelengths λ = 2 mm and λ = 1 mm clearly show evidence of parasitic capacitance through a measured offset. The precision of the shield’s distance in this measurement was ±1 µm. The uncertainty in the absolute distance is about ±15 µm due to mechanical factors. The accuracy and precision of the impedance analyzer (Hioki IM 3950) was about ±0.005 pF. The measurements were made at 50 Hz and each data point is an average of 4 cycles. A detailed analysis of this result is given in Section 3.3.

### 3.2. Choosing a Shield Distance

In free space, the electric field above the sensor decays smoothly [9,19]. Definitions for the effective electric field penetration depth have been made for different purposes and range from λ⁄2 to λ⁄(2π) [8,19,20,27]. For the purpose of making a quantitative comparison of the results incorporating the shield and the asymptotic C(s) behavior, a convention from Gaussian beam optics was borrowed. A definition of the distance for the shield has been made such that the capacitance is equal to $1\;-\;e^{-2}\;=\;86.5\text{\%}$ of the unshielded (s = ∞) capacitance value. That is, the distance $s_{0}$ of the shield fulfills the criterion:

(5) $${C(s}_{0})/C\left(s\;=\;\infty \right)=\;1\;-\;e^{-2}$$

The quantified results of the distance $s_{0}$ and its effect on the capacitance are shown in Table 2. One immediate outcome is that the shield distance $s_{0}$, satisfying Equation (5), varies strongly with the parasitic capacitance. In the absence of parasitic capacitance, this would be $s_{0}$ = 1.65λ. From calculations of the idealized geometry it is found that doubling this distance to 3.3λ gives 95% of the unshielded capacitance and half this distance (0.83λ) reduces this capacitance to 63.2%. 

### 3.3. Difference Between Experiment and Theory

**Figure 6:**
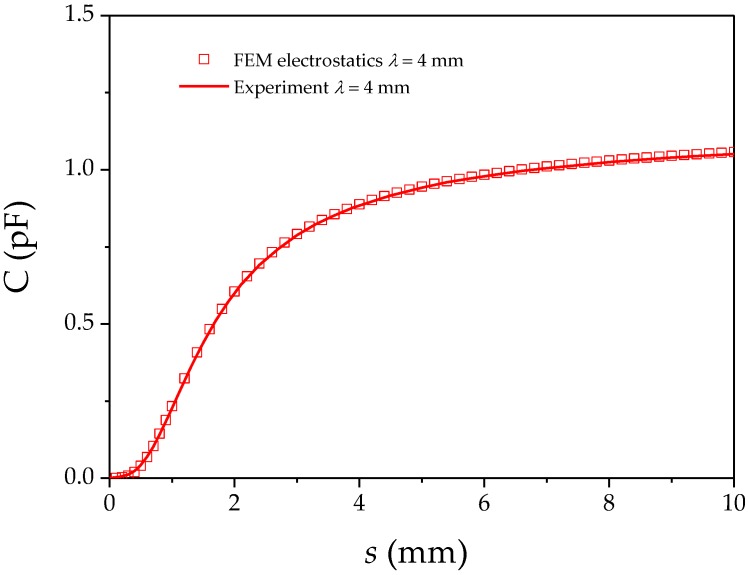
Simulated experiment based on the dimensions from the IDS cross sectional record via SEM and their permittivities. Incorporating these in FEM can accurately describe the practical environment.

In theory, the maximum capacitance achieved was for the 4 mm wavelength sensor and reached 0.7 pF. The experiment shows a considerable difference with an observed maximum of 1.35 pF. As the Green’s function result is consistent with the FEM solver, this difference can be explained as due to the simplifications made in the geometry for the calculation. Each sensor’s wavelength determines the spatial distribution of the emanated electric field. The dimensions of the insulator layer between the ground plane and electrodes is constant among all λ, but at smaller λ the ground plane is relatively further away and thus less effective at excluding flux from this region. Hence, the insulation layer $d_{\mathrm{insulator}}$ (FR4) was sensed and added to the total capacitance. For smaller wavelengths than λ = 4 mm this impact is measurable as a significant offset. In turn, the larger λ, the less the insulator thickness influences the sensing properties. These observations agree with the literature [8]. As a result, a higher ratio of λ/$d_{\mathrm{insulator}}$ moves the experiment closer to the theoretical geometry defined in Figure 2. A more realistic modeling in FEM (electrostatics module, Comsol Multiphysics 4.2) could accurately reproduce the measurement by incorporating the spatial structure shown as the cross sectional view in Figure 3c as shown for λ = 4 mm in Figure 6. 

**Figure 7:**
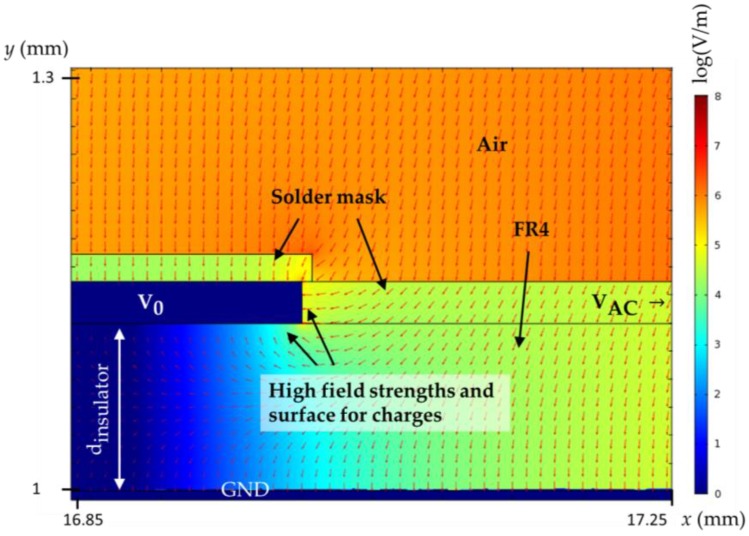
Simulated electric field around the neutral electrode $V_{0}$. The finite thickness of the electrode produces an additional surface directly opposite the active electrode causing charge accumulation. Electric field lines from above the electrodes flow furthermore into the insulator (FR4) above the ground plane and terminate at the underside of the electrode $V_{0}$. These effects result in increased and parasitic capacitance.

This simulation of the IDS with λ = 4 mm could then be used to explore the nature of the parasitic capacitance and magnitude of capacitance compared to theory. Figure 7 shows the FEM simulation of the section in Figure 3c. The solder mask in Figure 7 is an additional dielectric between the air and the electrode which contributes to the capacitance. More significantly, the highest field strength adjacent to the electrode occurs in the gap between the active and neutral electrodes. This is the region covered by the solder mask and offers a surface for charges which was approximated to have zero thickness in the calculations. Also, some field lines from the active electrode are directed below the neutral electrode which agrees with the qualitative findings in the literature [19]. Thus, a large amount of the capacitance is occurring within the insulator material surrounding the electrodes instead of within the air above the sensor. 

**Figure 8:**
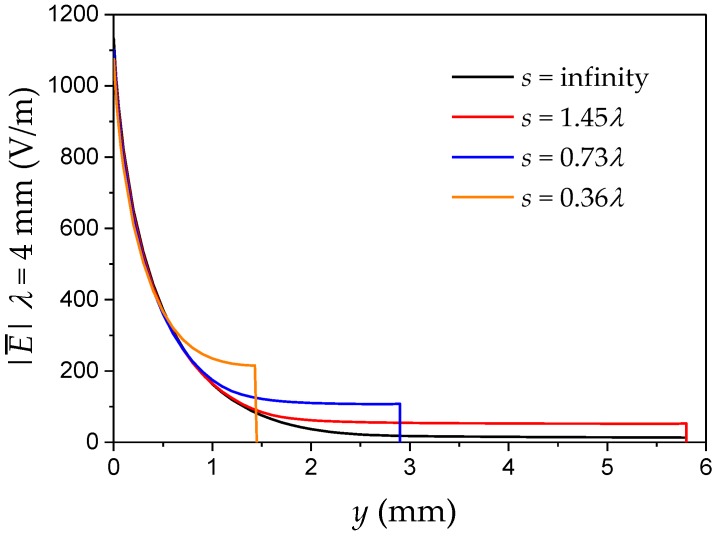
Average electric field strength |$\bar{E}$| across the IDS with λ = 4 mm, with a shield distance at 1.45λ = 5.8 mm, 0.73λ = 2.9 mm, 0.36 = 1.45 mm and at infinity^2^.

For λ = 4 mm and *V* = 1 V, the mean electric field strength across the sensor from Figure 6 could be plotted as a function of distance as shown in Figure 8. It shows the unshielded electric field and the shield position at $s_{0}$, $s_{0}$/2 and $s_{0}$/4. The initial field strength is about 1100 V/m which is due to the gap between electrodes of 1 mm. Clearly, the electric field decays smoothly with distance and with increasing shield position it approximates the unshielded field distribution (the FEM *y*-boundary for the infinite shield distance condition was at *s* = 25*λ*).

### 3.4. Validation Test of Shield’s Impact on Noise Reduction

**Figure 9:**
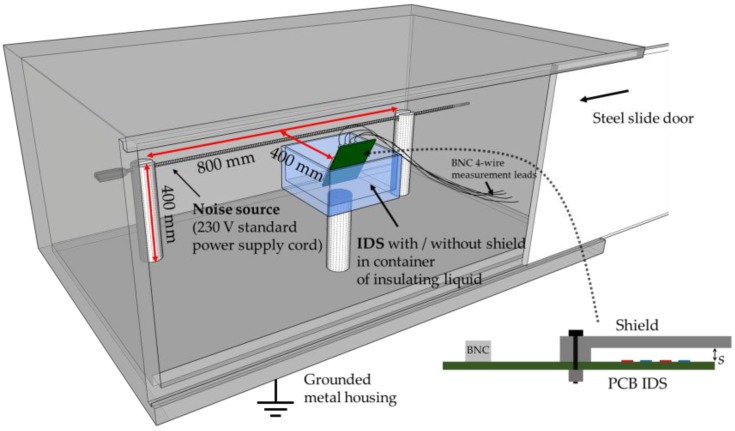
Experimental arrangement of the setup to test the function of the shield onto the IDS at distance $s_{0}$ = 1.5λ. Relevant dimensions are shown.

**Figure 10:**
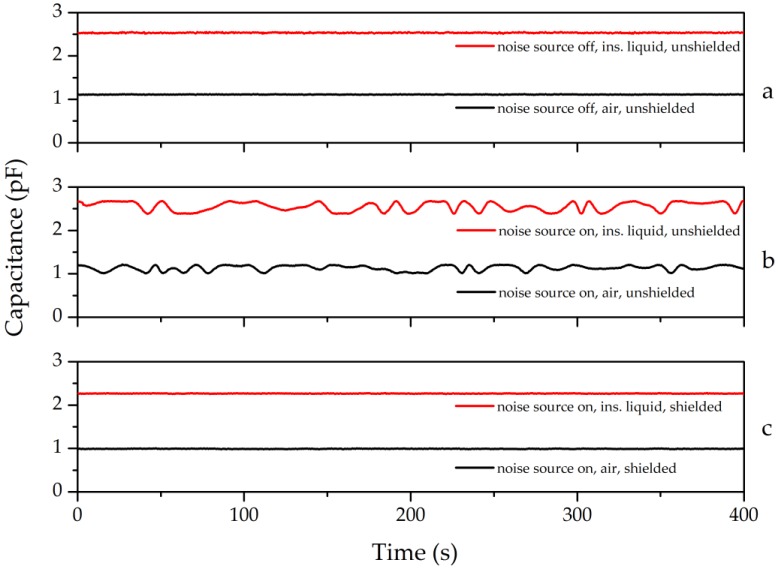
The validation of shield function for *λ* = 4 mm. (**a**) In the absence of an applied potential on the power supply cord, no instabilities are exhibited; (**b**) After applying voltage onto the cord, fluctuations are observed; (**c**) Measurement of capacitance with a Faraday shield in place.

In order to experimentally measure the effects of noise and shielding, a test using the largest wavelength sensor (λ = 4 mm) with a shield applied at $s_{0}=1.5\lambda =6\;\mathrm{mm}$ was conducted. A grounded and electrically conducting box shielded the IDS from external noise sources. This IDS was placed in a glass container, held mechanically 5 cm away from the glass and a standard power cord was arranged above the sensor’s surface as an electric noise source, as shown in Figure 9. The IDS was located 400 mm from the center of the 800 mm long power cord. The resulting electric field strength from this noise source onto the sensor is estimated to be ~500 V/m which is in the same order of magnitude of the IDS field, shown in Figure 8. The insulating liquids are measured at the line frequency [16], in the laboratory and experiment this frequency was 50 Hz. The test sequence comprised measurements without applied voltage into the cable and without IDS shield. Subsequently, the power cord was subject to 230 V and the IDS capacitance was measured once in air and once in an insulating liquid. Finally, the shield was applied and the test repeated. The results of the 801 recorded data points, collected at 0.5 s intervals, are shown in Figure 10.

**Figure 11:**
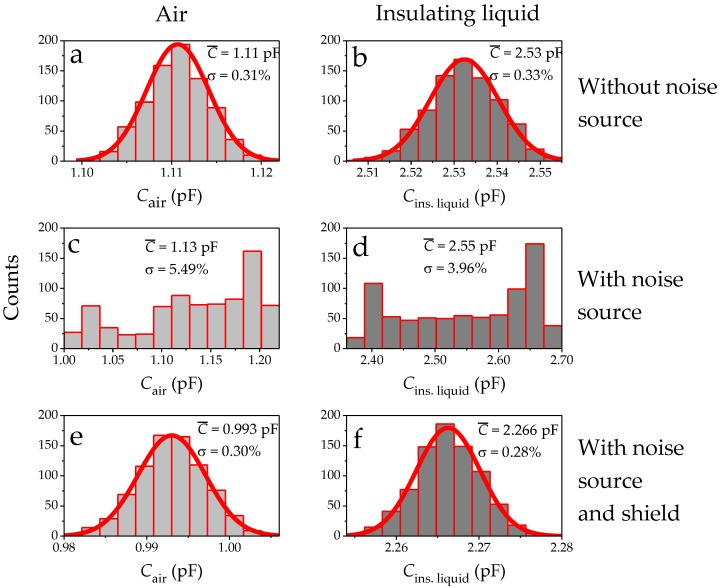
The capacitance values plotted as histograms. Capacitance values without the noise source for media of (**a**) air and (**b**) insulating liquid. Capacitance with the noise source for media (**c**) air and (**d**) insulating liquid. Capacitance with shield and noise source for media (**e**) air and (**f**) insulating liquid.

The measurements over 801 points are shown in Figure 10. When unshielded, the measurement exhibited a constant value. The noise is clearly increased when the power cord is subject to power which could be suppressed when the shield was applied. A quantitative examination is done in Figure 11.

The distribution of measured values from Figure 10 have been plotted in Figure 11. The capacitance values for the IDS with media air (Figure 11a) and insulating liquid (Figure 11b), without noise source and unshielded, exhibited a Gaussian distribution. The mean values were $\bar{C}$_air_ =1.11 pF (Figure 11a) and $\bar{C}$_ins liquid_ = 2.53 pF (Figure 11b), with small fluctuations resulting in *σ*_air_ and *σ*_oil_ ≤ 0.35%. This behavior changed when the noise source was turned on. With the noise source, $\bar{C}$_air_ and $\bar{C}$_ins. liquid_ increases by 0.02 pF (Figure 11c,d) while *σ* increases by a factor of 12–18 relative to the measurement without noise source. The distribution becomes flat and non-Gaussian. The spread of measurement values is due to the inherent frequency fluctuations of the IDS sensing frequency (50.000 ± 0.001 Hz) and the fluctuations of the grid (noise source) frequency (50.00 ± 0.01 Hz) causing an aperiodic interference pattern as can be seen in Figure 10b. With the 3D shield, the lower *σ* values and the observed Gaussian distribution in Figure 11a,b could be recovered (Figure 11e,f). The mean values $\bar{C}$_air_ and $\bar{C}$_ins. liquid_ have changed due to the impact of the shield on the IDS capacitance. This change of −11.5% is equal for the air (c.f. Figure 11a vs. Figure 11e) and insulating liquid (c.f. Figure 11b vs. Figure 11f) as media. In summary, this histogram analysis shows that the shield reduces the external noise effectively. 

In order to analyze how the measured permittivity is affected by noise and shield, Table 3 shows the calculated values for $\epsilon_{r}$ using the mean values from Figure 11. Mere averaging over 801 data points seems to be an incomplete method to recover the $\epsilon_{r}$ value obtained without the noise source. With the noise source, the difference in capacitance remained 0.02 pF affecting the sensed $\epsilon_{r}$ value. Due to error propagation, the standard deviation for $\epsilon_{r}$ is higher than for the capacitance. Determining $\epsilon_{r}$ of the insulating liquid with high certainty is therefore difficult; the standard deviation, *σ*, without the shield was as high as 9.5% whereas the shielded standard deviation of 0.6% is similar to the measurement without the noise source. 

The measured $\epsilon_{r}$ of insulating liquid with the used *λ* = 4 mm IDS ($\epsilon_{r}=$ 2.28) also agrees with literature values [17,18,28]. This would not have been the case for *λ* = 1 mm and *λ* = 2 mm due to the measured offset. 

## 4. Summary and Conclusions 

This research advances measuring $\epsilon_{r}$ of insulation liquids using IDS in the presence of external noise. It is shown under what conditions precise and more accurate measurements can be made. The dependence of the IDS capacitance on shield distance was predicted using a Green’s function method for mixed finite and infinite boundaries. FEM matched the obtained solution as a complementary measure. The manufactured interdigitated dielectrometry sensor differed geometrically from the simplified model which resulted in a difference between the Green’s function model and experimental results. Therefore, FEM was used to model a more realistic situation. An evaluation of these results showed a discrepancy between experimental and theoretical results, indicating that a smaller parasitic capacitance occurs the larger λ is relative to a constant thickness of insulator, $d_{\mathrm{insulator}}$, separating the electrodes and ground plane.

A parameter $s_{0}$ was defined as the distance for the shield which retained 86.5% of the capacitance. This ranged in the experiment from 0.65λ to 1.45λ. The theoretical value for the simplified model, 1.65λ, was only dependent on the sensor’s wavelength.

For a demonstration, a shield was manufactured and applied to measure the permittivity of an insulating fluid. A considerable reduction in noise is shown via an experiment with a power cord as noise source. Measuring the permittivity at 50 Hz with the unshielded IDS exhibited a measured standard deviation *σ* = 9.5% whereas with shield *σ* = 0.6%.

## Figures and Tables

**Table 1:** Measured dimensions of the fabricated sensor in a cross-sectional view. The permittivity of the soldermask and insulator layer have been determined to be 4.5 [25,26].

Component	Height	Permittivity
FR4 insulator ($d_{\mathrm{insulator}}$)	119.5 μm	4.5
Solder mask between electrodes	30 μm	4.5
Solder mask above electrode	20 μm	4.5
Electrode thickness	32 μm	-

**Table 2:** Values for the capacitance with shield at $s_{0}$ in experiment and theory for all three studied wavelengths.

Wavelength of IDS	Experimental Shield Distance $s_{0}$	Experimental Capacitance Shielded	Experimental Capacitance Unshielded	Theoretical Shield Distance $s_{0}$	Theoretical Capacitance Shielded	Theoretical Capacitance Unshielded
λ = 4 mm	5.8 mm = 1.45λ	0.97 pF	1.12 pF	6.5 mm = 1.65λ	0.59 pF	0.68 pF
λ = 2 mm	2 mm = λ	0.90 pF	1.04 pF	3.25 mm = 1.65λ	0.29 pF	0.34 pF
λ = 1 mm	0.65 mm= 0.65λ	1.16 pF	1.35 pF	1.62 mm = 1.65λ	0.15 pF	0.17 pF

**Table 3:** Influence of noise source and shield on permittivity for *λ* = 4 mm.

Measurement	Mean Value ^1^	Standard Deviation σ
$\epsilon_{r}$ unshielded without noise source	2.28	0.64%
$\epsilon_{r}$ unshielded with noise source	2.25	9.45%
$\epsilon_{r}$ shielded with noise source	2.28	0.58%

^1^. Mean value is the arithmetical average of 801 data points each taken at 0.5 s.

## References

[1] Fofana I. (2013). 50 years in the development of insulating liquids. IEEE Electr. Insul. Mag..

[2] Rafiq M., Lv Y.Z., Zhou Y., Ma K.B., Wang W., Li C.R., Wang Q. (2015). Use of vegetable oils as transformer oils—A review. Renew. Sustain. Energy Rev..

[3] Rouse T.O. (1998). Mineral insulating oil in transformers. IEEE Electr. Insul. Mag..

[4] Yu C.L., Moore R.A. (1968). Properties of alternately charged coplanar parallel strips by conformal mappings. IEEE Trans. Electr. Devices.

[5] Seaver A.E., Mamishev A.V., Du Y., Lesieutre B.C., Zahn M. (1999). Development and applications of fringing electric field dielectrometry sensors and parameter estimation algorithms. J. Electr..

[6] Cao S., Pyatt S., Anthony C., Kubba A., Kubba A., Olatunbosun O. (2016). Flexible Bond Wire Capacitive Strain Sensor for Vehicle Tyres. Sensors.

[7] Khan M., Kang S.-W. (2016). Highly Sensitive Temperature Sensors Based on Fiber-Optic PWM and Capacitance Variation Using Thermochromic Sensing Membrane. Sensors.

[8] Mamishev A., Yanqing D., Zahn M. Measurement of Dielectric Property Distributions Using Interdigital Dielectrometry Sensors. Proceeding of the Conference on Electrical Insulation and Dielectric Phenomena.

[9] Mamishev A.V., Cantrell S.R., Du Y., Lesieutre B.C., Zahn M. (2002). Uncertainty in multiple penetration depth fringing electric field sensor measurements. IEEE Trans. Instrum. Meas..

[10] Wang H., Chen L., Wang J., Sun Q., Zhao Y. (2014). A Micro Oxygen Sensor Based on a Nano Sol-Gel TiO_2_ Thin Film. Sensors.

[11] Fong C.-F., Dai C.-L., Wu C.-C. (2015). Fabrication and Characterization of a Micro Methanol Sensor Using the CMOS-MEMS Technique. Sensors.

[12] Khan M., Khalilian A., Kang S.-W. (2016). Fast, Highly-Sensitive, and Wide-Dynamic-Range Interdigitated Capacitor Glucose Biosensor Using Solvatochromic Dye-Containing Sensing Membrane. Sensors.

[13] Khan M., Khalilian A., Kang S.-W. (2016). A High Sensitivity IDC-Electronic Tongue Using Dielectric/Sensing Membranes with Solvatochromic Dyes. Sensors.

[14] Feng J., Kang X., Zuo Q., Yuan C., Wang W., Zhao Y., Zhu L., Lu H., Chen J. (2016). Fabrication and Evaluation of a Graphene Oxide-Based Capacitive Humidity Sensor. Sensors.

[15] Xu S., Wang P., Dong Y. (2016). Measuring Electrolyte Impedance and Noise Simultaneously by Triangular Waveform Voltage and Principal Component Analysis. Sensors.

[16] (2004). Standard Insulating Liquids Measurement of Relative Permittivity, Dielectric Dissipation Factor (tan δ) and d.c. Resistivity (IEC 60247:2004).

[17] Dervos C.T., Paraskevas C.D., Skafidas P., Vassiliou P. (2005). Dielectric characterization of power transformer oils as a diagnostic life prediction method. IEEE Electr. Insul. Mag..

[18] Jung J.W., Lim Y.B., Jung J.S., Yi G.H., Park H.Y. The Analysis of Dielectric Characteristics of Transformer Oil Suffering Ageing Treatment. Proceeding of the 2006 IEEE 8th International Conference on Properties & applications of Dielectric Materials.

[19] Li X.B., Larson S.D., Zyuzin A.S., Mamishev A.V. (2006). Design principles for multichannel fringing electric field sensors. IEEE Sens. J..

[20] Mamishev A.V., Sundara-Rajan K., Fumin Y., Yanqing D., Zahn M. (2004). Interdigital sensors and transducers. Proc. IEEE.

[21] Mamishev A.V., Lesieutre B.C., Zahn M. (1998). Optimization of multi-wavelength interdigital dielectrometry instrumentation and algorithms. IEEE Trans. Dielectr. Electr. Insul..

[22] Clague D.S., Wheeler E.K. (2001). Dielectrophoretic manipulation of macromolecules: The electric field. Phys. Rev. E.

[23] Philip M.M. (1953). Methods of Theoretical Physics.

[24] den Otter M.W. (2002). Approximate expressions for the capacitance and electrostatic potential of interdigitated electrodes. Sens. Actuators A Phys..

[25] Chirap A., Popa V. Insertion Loss Measurement of a Lowpass Microwave Filter Manufactured on FR4 Laminate. Proceeding of the 2016 International Conference on Development and Application Systems (DAS).

[26] Holzman E.L. (2006). Wideband measurement of the dielectric constant of an FR4 substrate using a parallel-coupled microstrip resonator. IEEE Trans. Microw. Theory Tech..

[27] Sheiretov Y., Zahn M. Dielectrometry Measurements of Moisture Dynamics in Oil-Impregnated Pressboard. Proceeding of 1994 IEEE International Symposium on, Electrical Insulation.

[28] Ten C.F., Fernando M.A.R.M., Wang Z.D. Dielectric Properties Measurements of Transformer Oil, Paper and Pressboard with the Effect of Moisture and Ageing. Proceeding of Conference on Electrical Insulation and Dielectric Phenomena (2007 Annual Report).

